# Dengue and Zika Virus Diagnostic Testing for Patients with a Clinically Compatible Illness and Risk for Infection with Both Viruses

**DOI:** 10.15585/mmwr.rr6801a1

**Published:** 2019-06-14

**Authors:** Tyler M. Sharp, Marc Fischer, Jorge L. Muñoz-Jordán, Gabriela Paz-Bailey, J. Erin Staples, Christopher J. Gregory, Stephen H. Waterman

**Affiliations:** 1 Division of Vector-Borne Diseases, National Center for Emerging and Zoonotic Infectious Diseases, CDC

## Abstract

Dengue and Zika viruses are closely related mosquitoborne flaviviruses with similar transmission cycles, distribution throughout the tropics and subtropics, and disease manifestations including fever, rash, myalgia, and arthralgia. For patients with suspected dengue or Zika virus disease, nucleic acid amplification tests (NAATs) are the preferred method of diagnosis. Immunoglobulin M (IgM) antibody testing can identify additional infections and remains an important tool for the diagnosis of these diseases, but interpreting the results is complicated by cross-reactivity, and determining the specific timing of infection can be difficult. These limitations are a particular challenge for pregnant women in determining whether Zika virus infection occurred during or before the pregnancy.

This report summarizes existing and new guidance on dengue and Zika virus diagnostic testing for patients with a clinically compatible illness who live in or recently traveled to an area where there is risk for infection with both viruses. CDC recommendations for screening of asymptomatic pregnant women with possible Zika virus exposure are unchanged. For symptomatic nonpregnant persons, dengue and Zika virus NAATs should be performed on serum collected ≤7 days after symptom onset. Dengue and Zika virus IgM antibody testing should be performed on NAAT-negative serum specimens or serum collected >7 days after onset of symptoms. For symptomatic pregnant women, serum and urine specimens should be collected as soon as possible within 12 weeks of symptom onset for concurrent dengue and Zika virus NAATs and IgM antibody testing. Positive IgM antibody test results with negative NAAT results should be confirmed by neutralizing antibody tests when clinically or epidemiologically indicated, including for all pregnant women. Data on the epidemiology of viruses known to be circulating at the location of exposure and clinical findings should be considered when deciding which tests to perform and for interpreting results.

Patients with clinically suspected dengue should receive appropriate management to monitor and treat shock and hemorrhage. Women with laboratory evidence of possible Zika virus infection during pregnancy and their infants should be evaluated and managed for possible adverse outcomes. Dengue and Zika virus disease are nationally notifiable conditions, and cases should be reported to public health authorities.

## Introduction

Dengue and Zika viruses are closely related mosquitoborne flaviviruses with similar transmission cycles, distribution throughout the tropics, and disease manifestations (*1*,*2*). Although the four dengue viruses are the predominant flaviviruses transmitted by *Aedes* mosquitoes in the tropics and subtropics, the recent emergence of Zika virus has complicated diagnostic testing considerations and interpretation. For patients with suspected dengue or Zika virus disease, nucleic acid amplification tests (NAATs) are the preferred method of diagnosis because they can provide confirmed evidence of infection and distinguish the specific virus (*3*,*4*). Immunoglobulin M (IgM) and neutralizing antibody testing also can be used to identify dengue and Zika virus infections, particularly in patients who present after viral nucleic acid is no longer detectable (*4*–*7*). However, dengue and Zika virus antibody testing is complicated by cross-reactivity that might preclude conclusive determination of which flavivirus is responsible for the person’s recent infection (*8*). Moreover, because flavivirus IgM antibodies are often detectable for months after an infection, determining the specific timing of infection can be difficult, especially among persons who live in or frequently travel to areas with risk for dengue or Zika virus infection (*5*,*9*–*11*). These limitations are a particular challenge for pregnant women in determining whether Zika virus infection occurred during or before the pregnancy.

Previous guidance provided diagnostic testing recommendations for pregnant and nonpregnant persons with possible Zika virus infection (*12*–*14*). Existing CDC recommendations for screening of asymptomatic pregnant women with possible Zika virus exposure are unchanged (*12*). This report updates CDC’s diagnostic testing guidance for patients, including pregnant women, with an illness clinically compatible with dengue or Zika virus disease and who reside in or recently traveled to an area where there is risk for infection with both dengue and Zika viruses.

## Purpose

These guidelines provide federal, state, territorial, and local health departments with recommended diagnostic algorithms and interpretation of test results for evaluation of dengue and Zika virus infection in patients with a clinically compatible illness and relevant epidemiologic exposure. These recommendations have incorporated lessons learned and feedback from public health and commercial laboratories regarding previously published guidelines (*12*,*13*,*15*,*16*). Because recommendations are intended for health departments, laboratories and health departments can adapt the recommendations according to local needs, resource availability, capacity for diagnostic testing, and epidemiologic conditions. Up-to-date information on areas where there is risk for dengue and Zika virus infection and ongoing outbreaks are available online (*17*,*18*).

## Methods

A work group comprising CDC epidemiologists, physicians, and laboratorians was convened in January 2018. The group reviewed data regarding the natural history of dengue and Zika virus infections and the resulting immune response. In addition, they evaluated evidence on the performance of diagnostic tests to detect and differentiate dengue and Zika virus infections. Primary data sources included published peer-reviewed studies identified through searches of PubMed (n = 97) and Medline (n = 276) and references cited in relevant articles (n = 4). Unpublished data also were considered, including package inserts for products submitted to the U.S. Food and Drug Administration (FDA) for approval (n = 4) or Emergency Use Authorization (n = 19), and data derived from diagnostic testing performed at CDC (*19*). Evidence evaluated included the sensitivity and specificity of NAAT and IgM antibody assays by day postonset of illness, concordance of NAAT and serologic test results, and frequency of confirmation of IgM antibody detection with virus-specific neutralizing antibodies. Testing recommendations might be updated when additional assays have been evaluated and are approved for routine use by FDA.

## Dengue and Zika Virus Epidemiology and Clinical Manifestations

Dengue and Zika viruses are transmitted by *Aedes* species mosquitoes, primarily *Aedes aegypti*, which are present throughout the tropics and subtropics (*1*,*2*). Infection with any of these viruses can result in an acute illness that includes fever, rash, myalgia, and arthralgia. Certain patients with dengue will progress to potentially fatal severe dengue, for which appropriate clinical management can reduce the case-fatality rate among hospitalized patients to <0.5% (*20*,*21*). The incidence of dengue has doubled each decade since 1990 such that, in 2013, an estimated 58 million symptomatic infections and 13,000 deaths occurred worldwide (*2*,*22*,*23*).

Zika virus was first isolated in Uganda in 1947. For the next 60 years, only sporadic cases were identified in Africa and Asia until 2007, when the first outbreak was recognized in Micronesia (*24*–*27*). During 2013–2015, approximately 30,000 suspected Zika virus disease cases were reported from French Polynesia and other Pacific islands. During 2015–2016, large outbreaks occurred throughout much of the Americas (*1*,*28*,*29*). During these recent outbreaks, new modes of transmission (e.g., congenital, perinatal, and sexual) and clinical manifestations (e.g., fetal loss, microcephaly, serious birth defects of the brain and eye, Guillain-Barre syndrome, other neurologic syndromes, and severe thrombocytopenia) (*1*,*29*,*30*) were identified.

## Dengue and Zika Virus Infections and Immune Response

Most dengue and Zika virus infections are asymptomatic (*2*,*27*,*31*). Among symptomatic persons, the incubation period from infection until disease onset is a few days to 2 weeks (*32*–*34*). Dengue and Zika virus RNA are likely to be detected in serum from approximately 2 days before to 1 week after illness onset (*4*,*5*,*35*,*36*). However, detection of Zika virus nucleic acid might be prolonged in some patients, especially pregnant women (*5*,*37*–*41*). Zika virus RNA also can be detected in other body fluids (e.g., whole blood, urine, saliva, amniotic fluid, semen, and breast milk), and some reports suggest that viral RNA might be found at higher levels or for longer duration in some of these specimens (*7*,*42*–*48*). Dengue virus nonstructural protein-1 (NS1) antigen also can be detected in serum with similar frequency and duration as dengue viral RNA (*35*).

IgM antibodies directed against dengue and Zika virus typically develop during the first week of illness (*4*,*5*,*7*); however, limited published data exist on the duration of IgM antibodies following dengue or Zika virus infection. Among adults with dengue virus infection in Taiwan, 71% (31 of 44) had IgM antibodies against the envelope protein detectable at 6 months after acute infection, and 46% (20 of 44) had detectable IgM at 12 months after onset (*49*). Among 266 patients from Brazil with confirmed dengue virus infection, >70% had IgM antibody against the NS1 protein detected for >90 days after illness onset (*49*,*50*). In one study of patients with symptomatic Zika virus infection, 87% (52 of 60) had detectable IgM antibodies >60 days after symptom onset (*5*). In another cohort study of persons with confirmed Zika virus infection, 73% (45 of 62) had detectable IgM antibodies at 12–19 months after acute illness (*51*). Data for closely related flaviviruses (i.e., West Nile and yellow fever viruses) indicate that IgM antibodies might be detectable in serum for months or years after initial infection (*9*–*11*).

Neutralizing antibodies develop shortly after IgM antibodies and consist primarily of IgG antibodies. Neutralizing antibodies persist for multiple years after flavivirus infections and usually confer long-lived immunity (*52*–*54*). In persons previously infected with or vaccinated against a flavivirus, subsequent infection with another flavivirus (i.e., secondary flavivirus infection) can result in both a diminished IgM response and a rapid increase in neutralizing antibodies against multiple flaviviruses, which might preclude conclusive determination of which virus was responsible for the person’s recent infection (*4*,*50*,*55*–*59*).

## Dengue and Zika Virus Diagnostic Testing

Dengue and Zika virus diagnostic testing employs both molecular and serologic methods; testing for dengue virus also includes detection of NS1 antigen. For patients with suspected dengue or Zika virus disease, molecular testing can provide confirmed evidence of infection, and NAATs can distinguish the specific virus. However, despite the high sensitivity and specificity of NAATs, both false-negative and false-positive results can occur (*3*,*60*–*64*).

With IgM antibody testing, false-positive results are more common than with NAAT and can occur because of nonspecific reactivity or cross-reactivity with other flaviviruses (e.g., West Nile, St. Louis encephalitis, Japanese encephalitis, or yellow fever viruses) (*4*,*6*,*8*). In addition, because IgM antibodies might be detectable for months or longer after infection, determining the specific timing of dengue and Zika virus infection can be difficult, especially among persons who live in or frequently travel to areas where the disease is endemic. These limitations of serologic testing are a particular challenge for pregnant women and attempts to determine whether Zika virus infection might have occurred during or before pregnancy. With decreased incidence and thus lower likelihood of Zika virus infection, a higher proportion of positive IgM antibody tests will be due to cross-reactivity with dengue or other flavivirus antibodies, a previous Zika virus infection, or false-positive results.

Plaque reduction neutralization tests (PRNTs) are quantitative assays that measure virus-specific neutralizing antibody titers for dengue, Zika, and other flaviviruses to which the patient might have been exposed (*56*,*65*). For diagnostic testing, CDC uses a PRNT with a 90% cutoff value titer ≥10 in serum and ≥2 in cerebrospinal fluid (the typical starting dilutions) to define positive specimens. PRNTs can resolve false-positive IgM antibody results caused by nonspecific reactivity and, in certain cases, can help identify the infecting virus. In primary flavivirus infections, a neutralizing antibody titer ≥4-fold higher than titers against other flaviviruses to which the person might have been exposed usually determines the specific infecting flavivirus. Recent findings indicate that neutralizing antibody titers might be able to differentiate dengue and Zika virus infections, particularly in specimens collected ≥3 months after illness onset (*66*). However, at 12–19 months after confirmed Zika virus infection, 17 (27%) of 62 persons in Florida still had neutralizing antibody titers that could not distinguish between dengue and Zika viruses (*51*). On the basis of previous flavivirus research and limited data specific to dengue and Zika viruses, the historic use of a four-fold higher titer by PRNT might not discriminate between dengue and Zika virus antibodies during the acute illness, especially following secondary flavivirus infections (*8*,*56*,*57*,*66*). Consequently, in areas with high prevalence of dengue and Zika virus infections, PRNT might not define the infecting virus for a significant proportion of cases (*8*). Therefore, such jurisdictions should make informed decisions about the utility of PRNT depending on the prevalence of dengue and Zika virus infection and observed performance of PRNT to confirm IgM test results.

Most state health departments and numerous commercial laboratories perform dengue and Zika virus diagnostic testing, and confirmatory testing is available through multiple state health departments and CDC. FDA has cleared three assays for the diagnosis of dengue: 1) a NAAT for use on serum and whole blood, 2) an enzyme immunoassay that detects dengue virus NS1 antigen in serum, and 3) an enzyme immunoassay that detects dengue virus IgM antibodies in serum (*63*,*66*,*67*). FDA has cleared one enzyme immunoassay to detect Zika virus IgM antibodies in serum (*68*). FDA also has issued Emergency Use Authorizations for multiple NAAT and other IgM antibody assays to diagnose Zika virus infection (*19*). Various Zika virus NAATs can be used on serum, plasma, whole blood, cerebrospinal fluid, urine, or amniotic fluid. Zika virus IgM antibody assays can be used variably on serum, plasma, whole blood, or cerebrospinal fluid. Efforts are ongoing to develop and validate serologic assays to reliably differentiate dengue and Zika virus infections and to distinguish recent and previous infections (*69*).

During a dengue outbreak, jurisdictions might elect to forego Zika virus testing in nonpregnant persons with suspected dengue. Similarly, during a Zika virus outbreak, jurisdictions might elect to forego dengue virus testing in nonpregnant persons with suspected Zika virus disease. In both scenarios, because of possible adverse outcomes, pregnant women should be tested by NAAT for evidence of infection with both dengue and Zika viruses and by serology for evidence of infection with the virus causing the outbreak. Patients for whom testing is unable to determine the infecting flavivirus should be clinically managed for possible dengue and, in pregnant patients, Zika virus infection because they might have been infected with either virus.

## Clinical and Epidemiologic Criteria for Testing

Dengue and Zika virus testing should be considered in a patient with a clinically compatible illness who lives in or recently traveled to an area where there is risk for infection with these viruses. Zika virus testing also should be considered in symptomatic patients who had sex with someone who lives in or recently traveled to those areas (*17*,*18*).

The U.S. surveillance case definitions for dengue and Zika virus disease include both clinical and diagnostic testing criteria. The case definition for dengue includes fever with one or more other characteristic finding (i.e., nausea, vomiting, rash, headache, retro-orbital pain, myalgia, arthralgia, positive tourniquet test, leukopenia, or a dengue warning sign) (*70*). For Zika virus disease, the surveillance case definition requires a clinically compatible illness with fever, rash, arthralgia, or conjunctivitis (*71*). For patients who live in or recently traveled to areas where there is risk for dengue and Zika virus infection, the broader clinical findings (i.e., fever, rash, arthralgia, or conjunctivitis) should be taken into account when considering testing for possible infection with either virus. Other infectious etiologies to consider in the differential diagnosis include chikungunya, malaria, rubella, measles, hepatitis A, parvovirus, adenovirus, enterovirus, leptospirosis, rickettsiosis, and group A streptococcal infections.

Pregnant women face potential complications from infection with either dengue or Zika virus (*72*). Dengue virus infection might increase the risk for maternal death or obstetric complications (e.g., hemorrhage, preeclampsia, eclampsia, and vertical transmission) during the peripartum period (*73*–*75*). Zika virus infection during pregnancy can result in fetal loss, microcephaly, or serious birth defects including structural abnormalities of the brain and eye (*30*,*72*).

## Recommended Testing for Symptomatic Nonpregnant Patients

**FIGURE 1:**
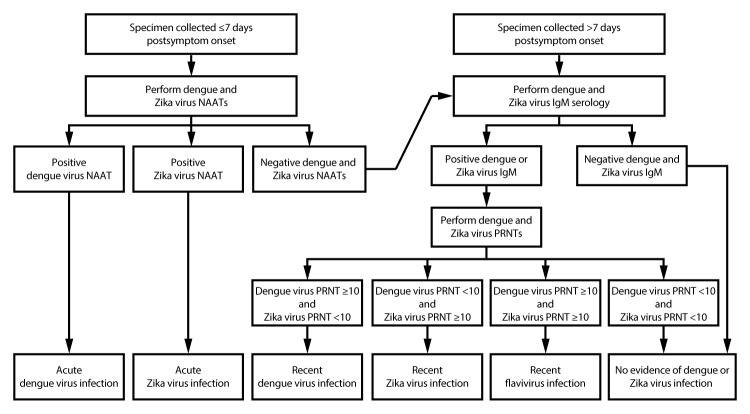
Dengue and Zika virus testing recommendations for nonpregnant persons with a clinically compatible illness and risk for infection with both viruses* **Abbreviations:** IgM = immunoglobulin M; NAAT = nucleic acid amplification test; PRNT = plaque reduction neutralization test. * *Specimen and test* *selection:* Dengue and Zika virus NAATs, IgM antibody testing, and PRNTs should be performed on serum. Some NAATs also can be performed on plasma, whole blood, cerebrospinal fluid, or urine, and some antibody tests can be performed on plasma, whole blood, or cerebrospinal fluid. Laboratories might choose to perform dengue and Zika virus NAATs and IgM antibody testing simultaneously rather than sequentially, or to perform dengue virus nonstructural protein-1 testing instead of dengue virus NAAT. *Indications to repeat assay(s):* If the patient’s illness has epidemiologic or clinical significance (e.g., first case of local transmission in area, new transmission mode, or unusual clinical syndrome), repeat a positive NAAT on newly extracted RNA from the same specimen. For indeterminate IgM antibody test results, repeat IgM antibody testing or perform PRNT on the same specimen. In areas where PRNTs are not performed, report the indeterminate results and request a second serum specimen for IgM antibody testing. *Interpretation of results:* Dengue and Zika virus IgM antibodies can be detected in serum for months following infection. The specific timing of infection cannot be determined. Data on the epidemiology of viruses known to be circulating at the location of exposure and clinical findings should be considered when interpreting the results of serologic diagnostic testing.

For nonpregnant persons with a clinically compatible illness, dengue and Zika virus NAATs should be performed on serum collected ≤7 days after symptom onset (Figure 1). Various NAATs also can be performed on plasma, whole blood, cerebrospinal fluid, or urine. For symptomatic persons with possible exposure to dengue and Zika virus, a positive NAAT result typically provides evidence of acute infection, and no antibody testing is indicated (Table). However, patients for whom the diagnosis has epidemiologic or clinical significance (e.g., first local transmission in area, new transmission mode, patient has an unusual clinical syndrome, or diagnosis will affect clinical management), a repeat NAAT should be performed on newly extracted RNA from the same specimen to rule out false-positive test results.

**TABLE:** Interpretation of dengue and Zika virus diagnostic test results for patients with a clinically compatible illness and risk for infection with both viruses

Dengue and Zika virus NAATs	Interpretation
Positive dengue virus assay, negative Zika virus assay	Acute dengue virus infection
Positive Zika virus assay, negative dengue virus assay	Acute Zika virus infection
Positive (both assays)	Acute dengue and Zika virus co-infection
Negative (both assays)	No evidence of dengue or Zika virus infection*

Dengue and Zika virus IgM antibodies	Dengue virus PRNT	Zika virus PRNT	Nonpregnant patients	Pregnant women
Positive (either assay)^†,§^	≥10	<10	Recent dengue virus infection	Dengue virus infection, timing cannot be determined
Positive (either assay)^†,§^	<10	≥10	Recent Zika virus infection	Zika virus infection, timing cannot be determined
Positive (either assay)^†^	≥10	≥10	Recent flavivirus infection^¶^	Flavivirus infection, timing cannot be determined
Any result	<10	<10	No evidence of dengue or Zika virus infection**	No evidence of dengue or Zika virus infection**
Positive dengue virus assay, negative Zika virus assay	Not performed		Presumptive recent dengue virus infection	Presumptive dengue virus infection, timing cannot be determined
Positive Zika virus assay, negative dengue virus assay	Not performed		Presumptive recent Zika virus infection	Presumptive Zika virus infection, timing cannot be determined
Positive (both assays)	Not performed		Presumptive recent flavivirus infection^¶^	Presumptive flavivirus infection, timing cannot be determined
Positive dengue virus assay, Zika virus assay not performed	Not performed		Presumptive recent flavivirus infection^¶^	Presumptive flavivirus infection, timing cannot be determined
Positive Zika virus assay, dengue virus assay not performed	Not performed		Presumptive recent flavivirus infection^¶^	Presumptive flavivirus infection, timing cannot be determined
Negative (both assays)	Not performed		No evidence of dengue or Zika virus infection**	No evidence of dengue or Zika virus infection**
Negative dengue virus assay, Zika virus assay not performed	Not performed		No evidence of dengue virus infection**	No evidence of dengue virus infection**
Negative Zika virus assay, dengue virus assay not performed	Not performed		No evidence of Zika virus infection**	No evidence of Zika virus infection**

**Abbreviations:** IgM = immunoglobulin M; NAATs = nucleic acid amplification tests; PRNT = plaque reduction neutralization test.

* In the absence of testing to detect IgM antibodies, negative NAAT results might reflect collection after clearance of detectable viral RNA and does not rule out infection.

^†^ Includes presumptive positive, indeterminate, and equivocal IgM antibody results that are not resolved by retesting.

^§^ IgM and PRNT antibody testing infrequently provide discordant results (e.g., dengue virus IgM positive, Zika virus IgM negative with dengue virus PRNT titer <10, Zika virus PRNT titer ≥10; or dengue virus IgM negative, Zika virus IgM positive with dengue virus PRNT titer ≥10, Zika virus PRNT titer <10). In such cases, report results as “presumptive flavivirus infection” and request a second specimen for retesting.

^¶^ The patient should be clinically managed for possible dengue and Zika virus infection because they might have been infected with either or both viruses. Data on the epidemiology of viruses known to be circulating at the location of exposure and clinical findings should be considered when interpreting the results.

** In the absence of NAAT, negative IgM or neutralizing antibody testing in specimens collected ≤7 days after illness onset might reflect collection before the development of a detectable antibody response and does not rule out infection.

Because of the decline in the level of viremia over time and possible inaccurate reporting of dates of illness onset, a negative NAAT result does not exclude dengue or Zika virus infection. Therefore, dengue and Zika virus IgM antibody testing should be performed on NAAT-negative serum specimens and serum collected >7 days after onset of symptoms. Certain IgM antibody assays also can be used on plasma, whole blood, or cerebrospinal fluid. Some laboratories might choose to perform dengue and Zika virus NAATs and IgM antibody testing simultaneously rather than sequentially.

For serum specimens collected <7 days after onset of symptoms, the combination of a negative NAAT result and negative IgM antibody testing suggests the patient did not have a recent flavivirus infection. However, in the absence of NAAT testing, a negative acute IgM antibody test might reflect specimen collection before development of detectable antibodies and does not rule out infection. For specimens collected from 7 days to 12 weeks after onset of symptoms, a negative IgM antibody result to both dengue and Zika virus rules out recent infection with either virus, and testing for other etiologies should be considered.

If either dengue or Zika virus IgM antibody testing is positive without a positive NAAT or NS1 antigen test, and definitive diagnosis is needed for clinical or epidemiologic purposes, confirmatory PRNTs should be performed against dengue, Zika, and other flaviviruses endemic to the region where exposure occurred. For indeterminate IgM antibody results, IgM antibody testing should be repeated on the same specimen or PRNTs performed. In the setting of positive IgM antibody testing, a PRNT titer ≥10 against dengue virus with negative PRNTs (i.e., <10) against Zika and other flaviviruses is considered evidence of recent dengue virus infection (Table). Conversely, a PRNT titer ≥10 against Zika virus with negative PRNTs against dengue and other flaviviruses is evidence of recent Zika virus infection. A PRNT titer ≥10 for both Zika and dengue virus (or another flavivirus) provides evidence of a recent infection with a flavivirus but precludes identification of the specific infecting virus or timing of infection on the basis of laboratory testing alone.

Negative PRNT titers against dengue and Zika virus in a serum specimen collected >7 days after illness onset rule out infection with either virus (Table). Without confirmatory PRNTs, determining whether positive IgM antibody results reflect a recent flavivirus infection or a false-positive result is not possible. In areas where PRNTs are not performed, positive dengue and negative Zika virus IgM antibody tests should be interpreted as a “presumptive recent dengue virus infection,” positive Zika and negative dengue virus IgM antibody tests should be interpreted as a “presumptive recent Zika virus infection,” and positive dengue and Zika virus IgM antibody tests should be interpreted as a “presumptive recent flavivirus infection.” If only one IgM antibody test was performed and was positive and PRNT was not performed, the second IgM test should be performed. If it is not possible to perform the second test, results of the first test should be interpreted as a “presumptive recent flavivirus infection.”

For nonpregnant patients who live in or recently returned from an area where there is an ongoing outbreak in which only one virus has been detected to be circulating, jurisdictions might reasonably elect to only perform testing for the virus causing the outbreak and not confirm IgM results by PRNT. If the patient does not reside in and has not recently traveled to an area with circulation of both viruses, and the only potential epidemiologic exposure is sexual contact with a person who has recently traveled to those areas, then the patient should only be tested for Zika virus infection.

## Recommended Testing for Symptomatic Pregnant Women

**FIGURE 2:**
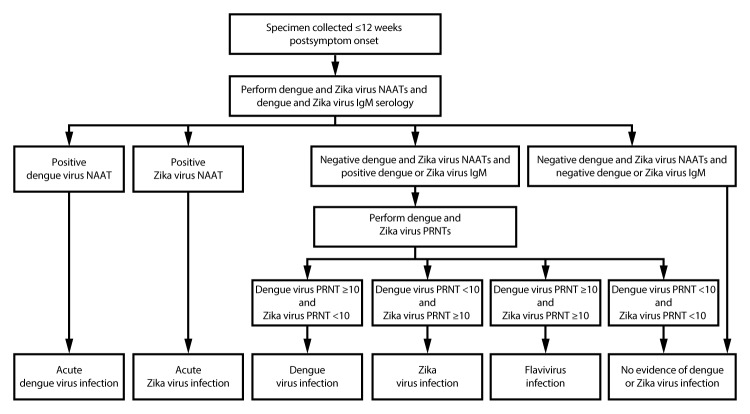
Dengue and Zika virus testing recommendations for pregnant persons with a clinically compatible illness and risk for infection with both viruses* **Abbreviations:** IgM = immunoglobulin M; NAAT = nucleic acid amplification test; PRNT = plaque reduction neutralization test. * *Specimen and test* *selection:* Dengue and Zika virus NAATs, IgM antibody testing, and PRNTs should be performed on serum. Some NAATs also can be performed on plasma, whole blood, cerebrospinal fluid, or urine, and some antibody tests can be performed on plasma, whole blood, or cerebrospinal fluid. Dengue virus NAAT does not need to be performed on specimens collected >7 days after illness onset. Some laboratories might choose to perform dengue virus nonstructural protein-1 testing instead of dengue virus NAAT. *Indications to repeat assay(s):* If Zika virus NAAT is positive on a single specimen but IgM antibody tests are negative, repeat NAAT on newly extracted RNA from the same specimen. For indeterminate IgM antibody test results, repeat IgM antibody testing or perform PRNT on the same specimen. In areas where PRNTs are not performed, report the indeterminate results and request a second serum specimen for IgM antibody testing. *Interpretation of results:* Dengue and Zika virus IgM antibodies can be detected in serum for months following infection. The specific timing of infection cannot be determined. Data on the epidemiology of viruses known to be circulating at the location of exposure and clinical findings should be considered when interpreting the results of serologic diagnostic testing.

Pregnant women with a clinically compatible illness and recent possible exposure to dengue and Zika virus should have concurrent diagnostic testing for dengue and Zika virus infection performed by NAAT and IgM antibody testing on a serum specimen and NAAT on a urine specimen to diagnose Zika virus infection (Figure 2). Specimens should be collected as soon as possible for dengue and Zika virus NAATs and within 12 weeks of symptom onset for Zika virus NAAT. Various NAATs also can be performed on plasma, whole blood, cerebrospinal fluid, or amniotic fluid; IgM antibody testing can be performed on plasma, whole blood, or cerebrospinal fluid. A positive NAAT result on any specimen typically provides evidence of recent infection. However, if NAAT is only positive for Zika virus on a single specimen and IgM antibody testing is negative, the NAAT should be repeated on newly extracted RNA from the same specimen to rule out false-positive test results.

If both dengue and Zika virus NAATs are negative but either IgM antibody test is positive, confirmatory PRNTs should be performed against dengue, Zika, and other flaviviruses endemic to the region where exposure occurred. For indeterminate IgM antibody results, IgM antibody testing should be repeated on the same specimen or PRNTs performed. If IgM antibody results are positive for one virus but the assay for the other virus was not performed, the second assay should be performed to appropriately interpret results. If the second assay is not performed, the single positive result should be interpreted as “presumptive flavirivus infection.” The interpretation for IgM and neutralizing antibody test results in a symptomatic pregnant woman is similar to that for nonpregnant patients; however, because dengue and Zika virus IgM antibodies can be detected in serum for months after an infection, the specific timing of infection cannot be determined, and other factors (e.g., epidemiologic and clinical findings) should be used to assess the likelihood that the current illness was due to either dengue or Zika virus and that exposure occurred during the pregnancy (Table). Pregnant women living in or returning from an area where there is an ongoing outbreak in which only one virus has been detected to be circulating should be tested by NAAT for evidence of infection with both dengue and Zika viruses; however, jurisdictions might elect to perform serologic diagnostic testing solely for the virus causing the outbreak.

Zika virus testing recommendations for asymptomatic pregnant women are unchanged (*12*). For asymptomatic pregnant women without ongoing risk for possible Zika virus infection, testing for Zika virus infection is not routinely recommended. However, testing should be considered using a shared decision-making model, in which patients and providers work together to make decisions about testing and care plans on the basis of patient preferences and values, clinical judgment, a balanced assessment of risks and expected outcomes, and the jurisdiction’s recommendations.

## Management of Patients with Dengue or Zika Virus Infection

Data on the epidemiology of viruses known to be circulating at the location of exposure and clinical findings should be considered in clinical and testing decisions and when interpreting results (*17*,*18*). All patients with clinically suspected dengue should receive appropriate management to monitor for shock and reduce the risk for complications resulting from plasma leakage and organ damage without waiting for diagnostic test results to be received (*20*). Women with laboratory evidence of possible Zika virus infection during pregnancy and their infants should be evaluated and managed for possible adverse outcomes, including congenital Zika virus infection (*12*,*76*). Patients for whom testing is unable to determine the most recent infecting flavivirus should be clinically managed for possible dengue and, in pregnant patients, Zika virus infection because they might have been infected with either virus. Health care providers with questions about test result interpretation should consult with state or local public health authorities for assistance.

## Reporting Dengue and Zika Virus Disease Cases

Dengue and Zika virus disease are nationally notifiable conditions. Health care providers are encouraged to report suspected dengue and Zika virus disease cases to their state, territorial, or local health departments to facilitate diagnosis and mitigate the risk for local transmission. State and territorial health departments should report cases to CDC according to the Council of State and Territorial Epidemiologists case definitions (*70**,**71*).
